# A novel miniature in-line load-cell to measure *in-situ* tensile forces in the tibialis anterior tendon of rats

**DOI:** 10.1371/journal.pone.0185209

**Published:** 2017-09-21

**Authors:** Martin Schmoll, Ewald Unger, Manfred Bijak, Martin Stoiber, Hermann Lanmüller, Jonathan Charles Jarvis

**Affiliations:** 1 Center for Medical Physics and Biomedical Engineering, Medical University Vienna, Waehringer Guertel, Vienna, Austria; 2 School of Sport and Exercise Sciences, Liverpool John Moores University, Liverpool, United Kingdom; Universite de Nantes, FRANCE

## Abstract

Direct measurements of muscular forces usually require a substantial rearrangement of the biomechanical system. To circumvent this problem, various indirect techniques have been used in the past. We introduce a novel direct method, using a lightweight (~0.5 g) miniature (3 x 3 x 7 mm) in-line load-cell to measure tension in the tibialis anterior tendon of rats. A linear motor was used to produce force-profiles to assess linearity, step-response, hysteresis and frequency behavior under controlled conditions. Sensor responses to a series of rectangular force-pulses correlated linearly (R^2^ = 0.999) within the range of 0–20 N. The maximal relative error at full scale (20 N) was 0.07% of the average measured signal. The standard deviation of the mean response to repeated 20 N force pulses was ± 0.04% of the mean response. The step-response of the load-cell showed the behavior of a PD2T2-element in control-engineering terminology. The maximal hysteretic error was 5.4% of the full-scale signal. Sinusoidal signals were attenuated maximally (-4 dB) at 200 Hz, within a measured range of 0.01–200 Hz. When measuring muscular forces this should be of minor concern as the fusion-frequency of muscles is generally much lower. The newly developed load-cell measured tensile forces of up to 20 N, without inelastic deformation of the sensor. It qualifies for various applications in which it is of interest directly to measure forces within a particular tendon causing only minimal disturbance to the biomechanical system.

## Introduction

Investigating the contractile properties of muscles to understand their function is a well-established field of research. Measurement of force allows the quantitative assessment of strength, endurance and fatigue. The hind limb muscles of various laboratory animals have been widely used as experimental models [1–9]. These muscles are relatively large and have a single tendon of insertion. Their nerves, which can be stimulated to activate the muscles via the motor end plates, are relatively easy to access and to isolate. A number of techniques have been developed to measure muscular forces in anaesthetized animals.

One common technique is to restrict movement at the muscle´s origin appropriately (e.g. by fixing the associated bone) and attaching its tendon of insertion to a fixed force transducer or instrumented lever [1,4,5,10–12]. This method is also applicable to rather small muscles like the rat extensor digitorum longus muscle [1], but requires a considerable rearrangement of the in-vivo biomechanical system, because the muscle force is arranged to act against an immovable reference frame.

A less invasive approach has also been used [3,7], in which the intact animal is placed in an external fixation apparatus to determine contraction strength by measuring the torque generated to rotate the related joint, again with immobilization of one part of the skeleton. An positive aspect of this type of measurement is that the original direction of force transmission is not altered. A drawback, on the other hand, is the loss of selectivity because the measured torque is usually the summation of the action of several muscles. If force in a particular muscle or tendon is to be measured, it must be back-calculated by taking into account the biomechanical environment including estimates for the length of the effective moment arms and the center of rotation.

The use of an in-line force transducer offers the advantage that the force transmitted through a particular tendon can be measured while the muscle retains its physiological position. Buckle transducers, moving towards this principle, have previously been used in cats [13], wallabies [14], pigeons [15] and guinea fowls [16] to measure the tendinous loading of multiple plantar-flexors simultaneously. A buckle transducer is placed around the intact tendon, and therefore does not measure the tensile force in the tendon directly. Rather, it gives an estimate of transmitted load from the lateral bending forces generated within the transducer. The measured strain in the transducer is thus only an indirect estimate of the linear tension borne by the tendon. The position of the tendon within the transducer influences the measured force, and the material properties of the tendon itself influence the transmitted strain. Buckle style transducers can experience problems due to impingement, when being pressed against a hard surface (e.g. bone) which potentially alters the measurement characteristics of the sensor. For bigger tendons, this problem can be circumvented by using an implantable force transducer [17] which is placed inside the specimen.

Another technique for measuring tensile forces is the use of optical fibers inserted transversely through the tendon of interest. This method takes advantage of the change in geometric properties due to compressive forces produced by the surrounding tendon, which in turn modulate the intensity of light transmitted through the optic fibre [18]. Its minimally invasive quality encourages the use of this technique to investigate tensile forces during standard movement tasks in humans [19,20]. However, although there is a linear relationship between the applied tensile force and the measured electrical signal of optical fibre transducers, application specific calibration in-vivo is required to obtain absolute values. This is necessary, as the optical fibre is only able to detect compressive forces directly, which are proportional to the tensile strains by a complex relationship depending on the mechanical properties of the tendon (e.g. elasticity). Thus calibration data is valid only for a single case, and it is not possible to perform formal testing of the transducer system alone.

Our objective was to characterize a newly developed in-line load-cell by assessing its linearity, step-response, hysteresis and frequency behavior. The load-cell was designed to estimate the type and amount of loading acting on the tibialis anterior (TA) muscle in intact rats, during electrical nerve stimulation by means of implantable pulse generators. It allows for direct measurements of tensile forces during acute experiments under anesthesia.

In adult rats, the length of the freely accessible part of the TA tendon is about 9.4 mm [21], limiting the overall length of such a load-cell to 7 mm in order to cause minimal disturbance to the biomechanical system. For isometric tetanic contractions of the TA in healthy adult rats, peak force values have been reported [4] ranging from 12.38 N ± 0.82 N to 13.5 ± 0.21 N (mean ± standard error). In order to measure also the forces generated during eccentric contraction the device should be strong enough to withstand repeated peak forces up to 20 N, while being accurate enough to quantify the twitch response to a single electrical stimulation pulse typically ranging from 1.4 N [6] to 2.3 N [9]. We assume that in a practical set up an accuracy of ± 10% and a precision expressed by a coefficient of variation less than ± 5% is sufficient to estimate the amount of loading of the TA within the intact rat. To allow unhindered movements of the foot of the anaesthetized rat, the load-cell requires smooth edges and a light-weight construction of less than 1 g, which is the gravitational equivalent of ~0.5% of a 2 N single twitch. Operation in a moist environment as well as the need to clean it for reuse, makes a water-proof design essential.

## Material and methods

### The load-cell

**Fig 1:**
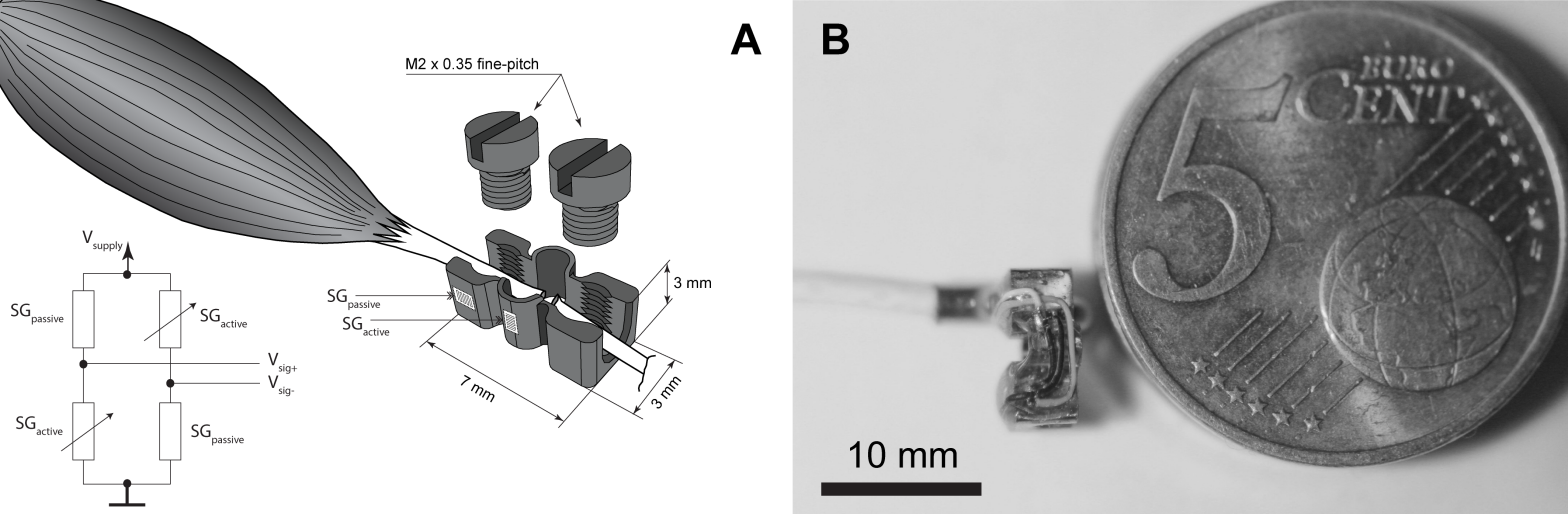
Working principle of the load-cell. A: Schematic representation of the clamping mechanism of the load cell. The illustration shows the dimensions of the load-cell as well as the position and orientation of the strain-gauges (SG) of the front side. The SGs on the back-side are positioned and orientated analogously. The circuit in the lower left corner shows the connection of the SG´s as temperature compensated half-bridge B: Manufactured load-cell in comparison to a 5 Euro-cent coin without clamping screws.

The newly developed load-cell (Center for Medical Physics and Biomedical Engineering, Medical University Vienna, Austria) has a CNC manufactured stainless steel core (3 x 3 x 7 mm) incorporating four strain gauges (1-LY11-0.3/120, Hottinger Baldwin Messtechnik GmbH, Darmstadt, Germany) operating as a temperature compensated half bridge (see Fig 1). The strain gauges were attached with cyanoacrylate glue (EP310S, Hottinger Baldwin Messtechnik GmbH, Darmstadt, Germany) and sealed with instant adhesive (Loctite 4850, Henkel Ltd, Herts, United Kingdom). This was necessary to improve the mechanical stability during handling and to allow for operation in a humid environment. To attach the tendon of interest to the load-cell, two M2 x 0.35 fine-pitch threaded screws were used. The resulting overall mass of the load-cell, including the clamping screws, was ~0.5 g. To make a longer part of the TA tendon freely available, the retinaculum was transected. Although the range of movement of the ankle joint was not affected by this procedure, the slightly altered pathway of force transmission needs to be considered as technical limitation. After clamping the load-cell to the intact tendon, the tendon is then cut through a small window between the screws. To mechanically strengthen the interface between the tendon and the clamps we allowed this part of the tendon to stiffen by drying for about 30 min until it became transparent, before full loading. The load-cell did not impede the shortening of the muscle nor the dorsiflexion of the foot. The connecting wires supported the load-cell, keeping it in position and avoided twisting or bending of the tendon.

### Experimental setup

#### Animals

All measurements were performed using the tibialis anterior tendons of a male adult Wistar rat from our local animal unit. Both tendons were harvested post-mortem directly after euthanasia (cervical dislocation without recovery from isoflurane anaesthesia) and stored in physiologic saline solution at 4°C until the next day. All actions were conducted in strict accordance to the Animals (Scientific Procedures) Act of 1986 and approved by the British Home Office (PPL 40/3280).

#### Bench testing of the load-cell

A range of force profiles to be applied to the load-cell were produced by a linear motor (Bose LM1 Testbench linear actuator, Bose Corporation, Framingham, Massachusetts, United States) and measured independently with a calibrated force sensor (50 lbf, Model: WMC-50-543, Bose Corporation, Framingham, Massachusetts, United States), the reference sensor.

**Fig 2:**
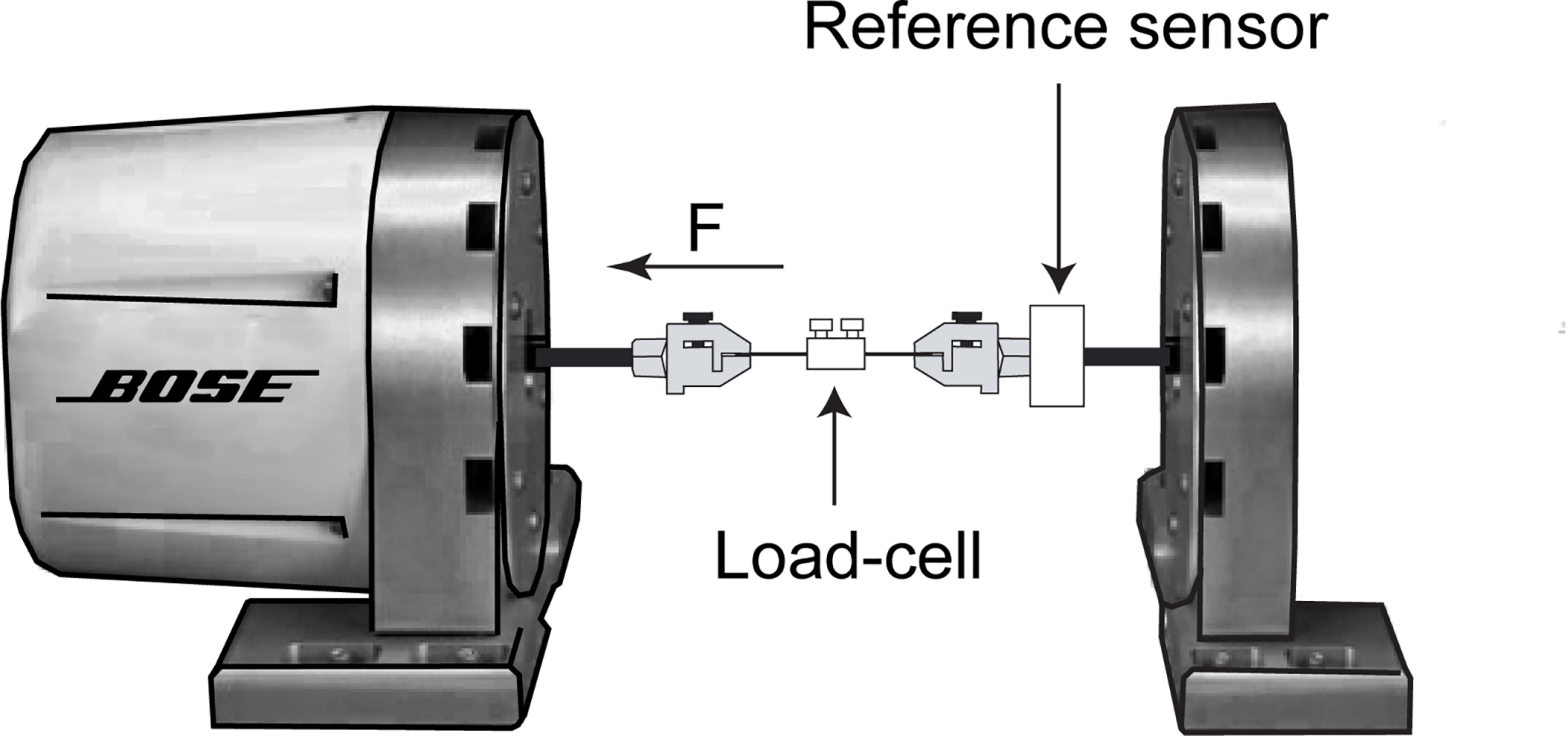
Measurement setup. A Bose linear motor applied preprogrammed patterns of force to the newly developed load-cell. The force measured with the reference sensor was used to correlate the output voltage of the load-cell with the load applied. The load-cell was tested using a dried tibialis anterior tendon of a rat which connected the device to the measurement set up.

Fig 2 shows an illustration of the measurement setup. The tendon of the tibialis anterior of a rat was used to connect both ends of the newly developed load-cell to the force-measurement system. The tendon at the clamping interface was dried for about 30 min after attachment to the load-cell until it became stiff and transparent as is our practice for *in-situ* measurements under terminal anesthesia.

#### Reproducibility

The reproducibility (practical accuracy and precision) was tested for the middle of the measurement range with a weight of 1 kg (9.81 N) on 5 consecutive days. The weight was attached to the load-cell using a nylon coated stranded stainless-steel wire (7 strands, loading capacity 12 kg, Balzer GmbH, Wartenberg, Germany) with an overall diameter of 0.55 mm. The coating was removed at the clamping points to avoid slipping. The signal of the load-cell was measured while applying the weight 5 consecutive times. The entire measurement configuration was set up and dismantled each day.

#### Data recording

The load-cell´s measurement bridge was connected to a PowerLab 26T digital recorder (ADInstruments Inc., Colorado Springs, USA) which provided the bridge voltage (0.562 V) and sampled the output voltage of both force and reference sensors at a sample rate of 20 kS / s with a resolution of 625 nV (in a 20 mV measuring range). Data recording, storing, pre-processing and exporting was done with ADInstruments LabChart 7 Pro installed on a standard personal computer (MSI GS60 2PE Ghost Pro, Micro-Star International, Zhonghe District, Taiwan).

### Tendon shrinkage measurement

To evaluate potential length changes caused by the drying process of the tendon, the amount of shrinkage was measured. One TA tendon was attached to the force generator without the load-cell. The initial length of the fresh and hydrated tendon between the metal-clamps was 8.2 mm. The force generator applied a constant force of 0.5 N for 75 min while the length of the tendon was constantly measured at a sample rate of 20 S / s.

### Bench testing protocol

All measurements were performed with a preload (offset) of 1 N, except measurements testing the frequency response which used a preload of 10 N. The preload avoided any sag of sensor and tendon to guarantee seamless transmission of force generated by the linear-motor to the load-cell in this bench test.

#### Calibration curve

**Fig 3:**
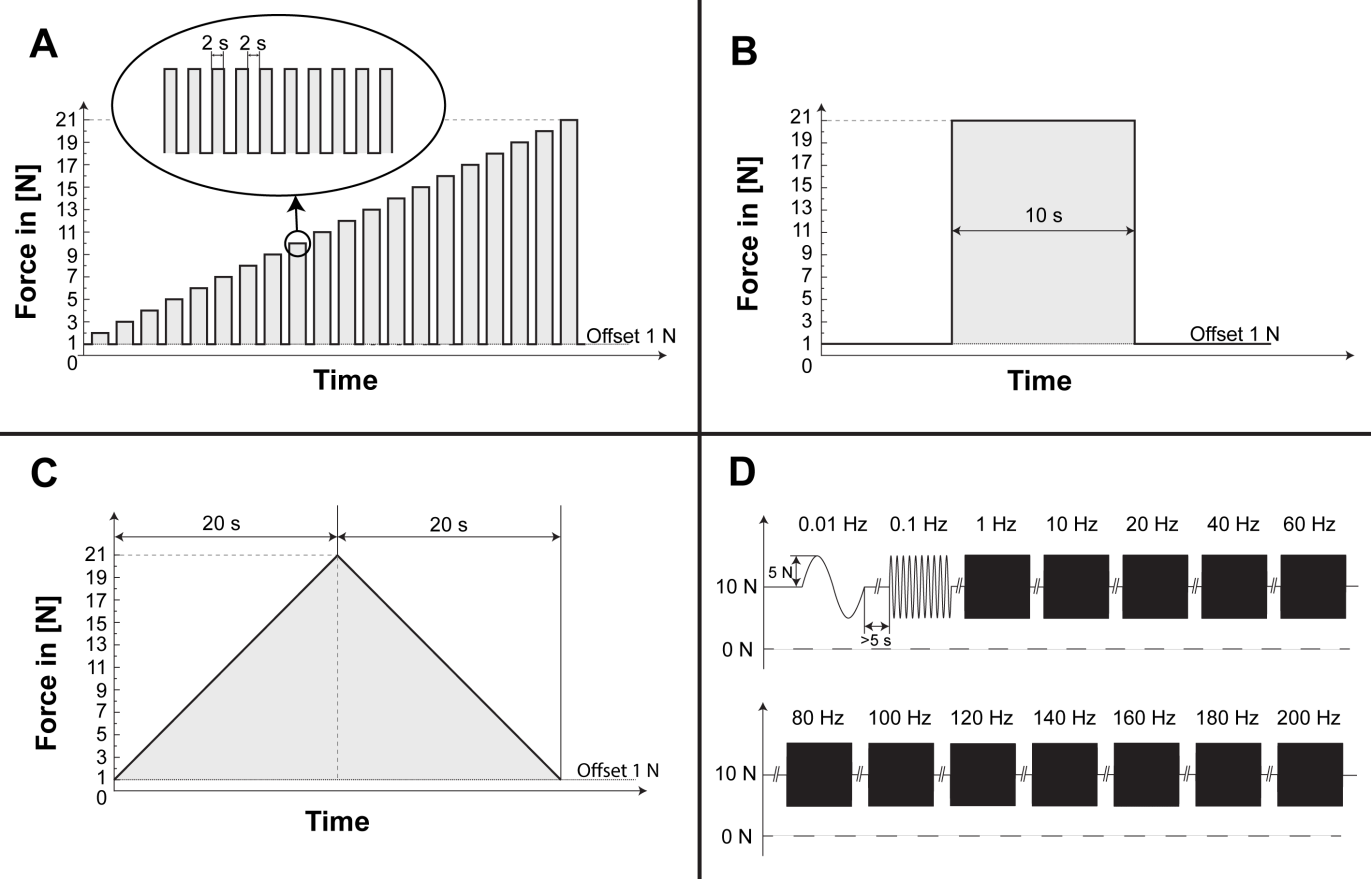
Testing protocol. Applied patterns of force to evaluate the behavior of the load-cell. A) Rectangular force-pulses of different amplitude–used to obtain calibration data. B) Rectangular force-step for evaluation of rapid changes. C) Triangular force-pattern to investigate hysteresis of the load-cell. D) Sinusoidal force-patterns of various frequencies to characterize the frequency behavior of the load-cell.

A calibration curve was calculated to correlate a measured change in output voltage of the load-cell with a certain change in applied force. Rectangular force pulses as illustrated in Fig 3A with a duration of 2 s were presented to the load-cell every 4 s. The amplitude of the pulses was increased after every tenth pulse by 1 N until an amplitude of 20 N was reached. The linearity of the calibration curve was assessed to test that the specified measurement range did not cause inelastic deformation of the sensor.

#### Step response

The step response characterizes the ability of a sensor to follow sudden changes of a particular input signal. The behavior of the load-cell was investigated by applying a rectangular force pulse with an amplitude of 20 N and a duration of 10 s as depicted in Fig 3B.

#### Hysteresis

The hysteresis describes the measured signal, when approaching a particular force-level either from the upper or lower end of the measurement range. This characteristic was evaluated by linearly increasing the force acting on the load-cell from 1 to 21 N over a duration of 20 s followed by a decrease to 1 N over the next 20 s. This triangular force-pattern, as shown in Fig 3C, was repeated over 4 consecutive cycles.

#### Frequency response

The frequency response specifies the amount of signal damping that can be expected for particular frequency components. Sinusoidal force bursts were applied with frequencies of 0.01, 0.1, 1, 10, 20, 40, 60, 80, 100, 120, 140, 160, 180 and 200 Hz (maximal frequency of the linear motor) as shown in Fig 3D. The force signal had an offset of 10 N, a peak amplitude of 5 N and was applied for a duration of at least 5 s.

### Data analysis

LabChart was used to manually set markers within the data, indicating the start and end of a particular response. This annotated data was exported for further processing with Matlab R2010a (MathWorks, Natick, Massachusetts, United States). Scripts were used to evaluate the output-voltage of the load-cell, with respect to the applied pattern of force. Those results were further processed and charted with Microsoft Excel (Microsoft Corporation, Washington, United States).

#### Calibration curve

All recorded voltage signals of the load-cell were normalized to the bridge voltage of 0.562 V, which is close to the maximum specified bridge voltage for the strain gauges of 0.6 V. A Matlab script automatically detected offset voltage V_offset_ (the average of 50 ms directly before the rising slope) and the maximal voltage V_max_ (the average of 50 ms directly before the falling slope) of the load-cell´s output, for every applied force pulse. The difference V_diff_ = V_max_−V_offset_ was considered to be the change in output voltage related to a rectangular force pulse of a certain magnitude. Mean ($\bar{V_{\mathrm{diff}}}$) and standard deviation was calculated from 10 consecutive pulses applied at the same force level. Further, the relative difference from $\bar{V_{\mathrm{diff}}}$ was evaluated for the minimum and maximum measured V_diff_.

#### Step response

The individual offsets were subtracted from the voltage signal of the load-cell and from the force-signal of the reference sensor. The resulting signals were normalized with respect to their particular maximal value, to allow comparison. In terms of control engineering, the response of the load-cell can be modeled as an additive combination of a P-element (proportional relationship, see Eq 1) with two PT1-elements (exponential relationship, see Eq 2), as defined in Eq 3, assuming operation within the linear (elastic) domain of the load-cell. Fitting was achieved by applying the normalized force-data (F_norm_) to the load-cell model (Eq 4), using Matlab´s function nlinfit to determine the parameters for each individual element.

$$P(t)=k_{0}$$ Eq 1

$$PT1(t)=k*\left(1-e^{-\frac{t}{T1}}\right)$$ Eq 2

$$LC(t)=P(t)+{PT1}_{1}(t)+{PT1}_{2}(t)$$ Eq 3

$$U_{fitted}(t)=F_{norm}(t)*LC(t)$$ Eq 4

These parameters were used to investigate the overall transfer-function in the frequency domain using a Laplace-transformed function of the load-cell model, as given in Eq 5. Simplification of Eq 5 revealed that the load-cell can be described with control engineering terminology [22] as PD2T2 element. The parameters of the PD2T2 element, were calculated using the values from Eq 4, resulting from the fitting process.

$$G_{LC}(s)=k_{0}+\frac{k_{1}}{1+s*{T1}_{1}}+\frac{k_{2}}{1+s*{T1}_{2}}$$ Eq 5

$$G_{PD_{2}T_{2}}(s)=k_{P}*\frac{\left(1+s*T_{A})*(1+s*T_{B}\right)}{\left(1+s*T_{C})*(1+s*T_{D}\right)}$$ Eq 6

#### Hysteresis

Four responses of the triangular waveform were superimposed and plotted in relation to the corresponding force registered by the reference sensor. The offsets (signal before the triangular force pattern started) were subtracted from the signals of the load-cell and the reference-sensor, and the maximal width of the hysteresis loop was determined.

#### Frequency response

The peak-to-peak amplitude, measured with the load-cell (V_PP_) and the reference sensor (F_PP_), was evaluated for every single period within a manually selected range, for each burst applied. Mean values of V_PP_ and F_PP_ were calculated for each single frequency and expressed in dB referring to the respective mean peak-to-peak value at 0.01 Hz.

$$V_{dB}=20*{log}_{10}\left(\frac{V_{PP}}{V_{ref}}\right)$$ Eq 7

$$F_{dB}=20*{log}_{10}\left(\frac{F_{PP}}{F_{ref}}\right)$$ Eq 8

#### Reproducibility

The change in output voltage of the load-cell was determined 2 s after applying a 1 kg (9.81 N) weight, the same way as described for the calibration curve. Mean ($\bar{V_{\mathrm{diff}}}$) and standard deviation of 5 consecutive measurements was calculated for each day.

## Results

The new load-cell was able to repeatedly measure tensile forces up to 21 N. The results show that this direct measurement technique is able to quantify the tensile forces transmitted by the TA tendon in rats.

### Tendon shrinkage

**Fig 4:**
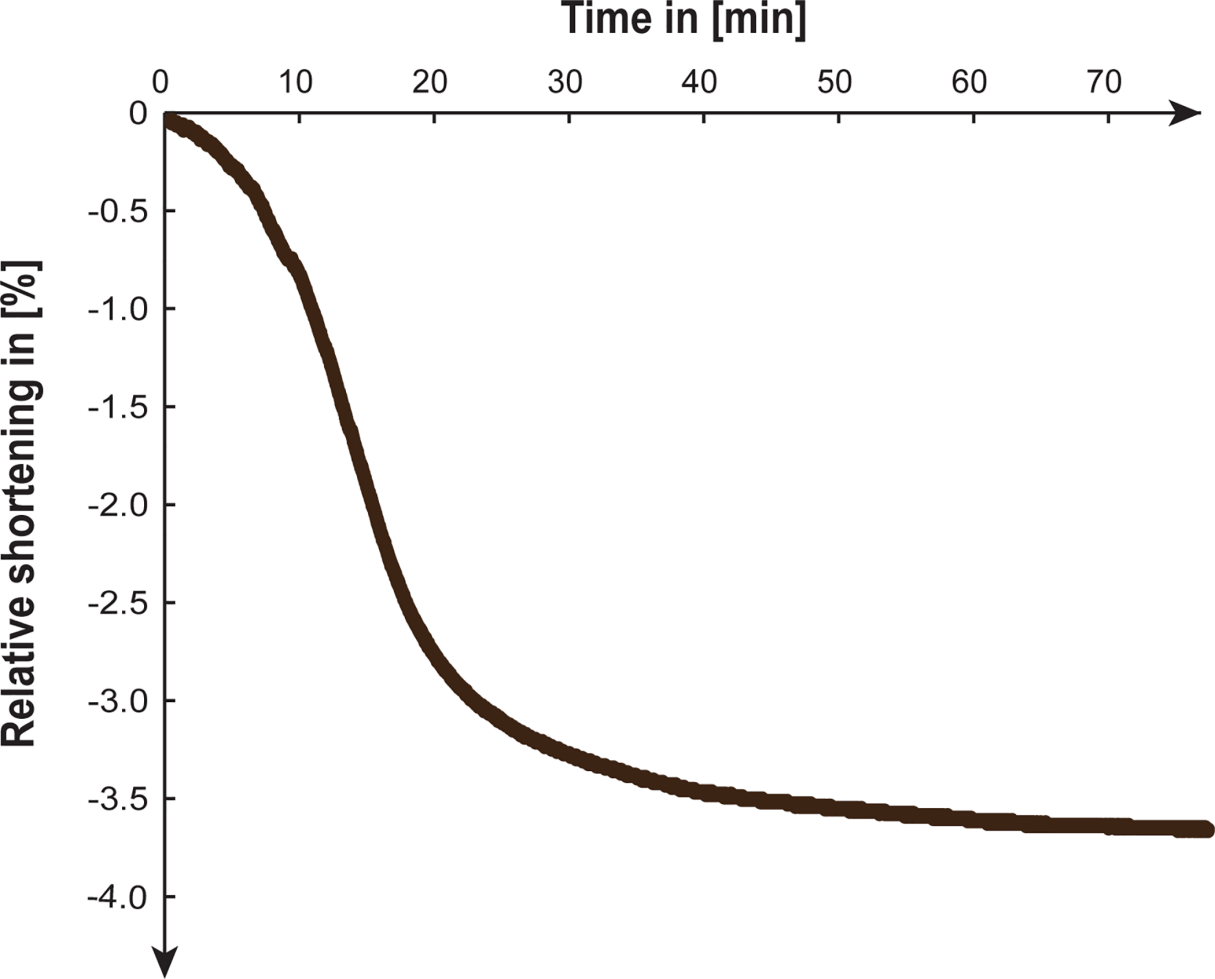
Tendon shrinkage. Measurement of the relative shortening during drying of the tibialis anterior tendon of a rat.

Fig 4 illustrates the relative shortening of a tibialis anterior tendon while drying for 75 min loaded with 0.5 N. The dry tendon was 0.3 mm shorter than the hydrated one, which correlates to a maximal relative shortening of 3.6%.

### Calibration curve

**Fig 5:**
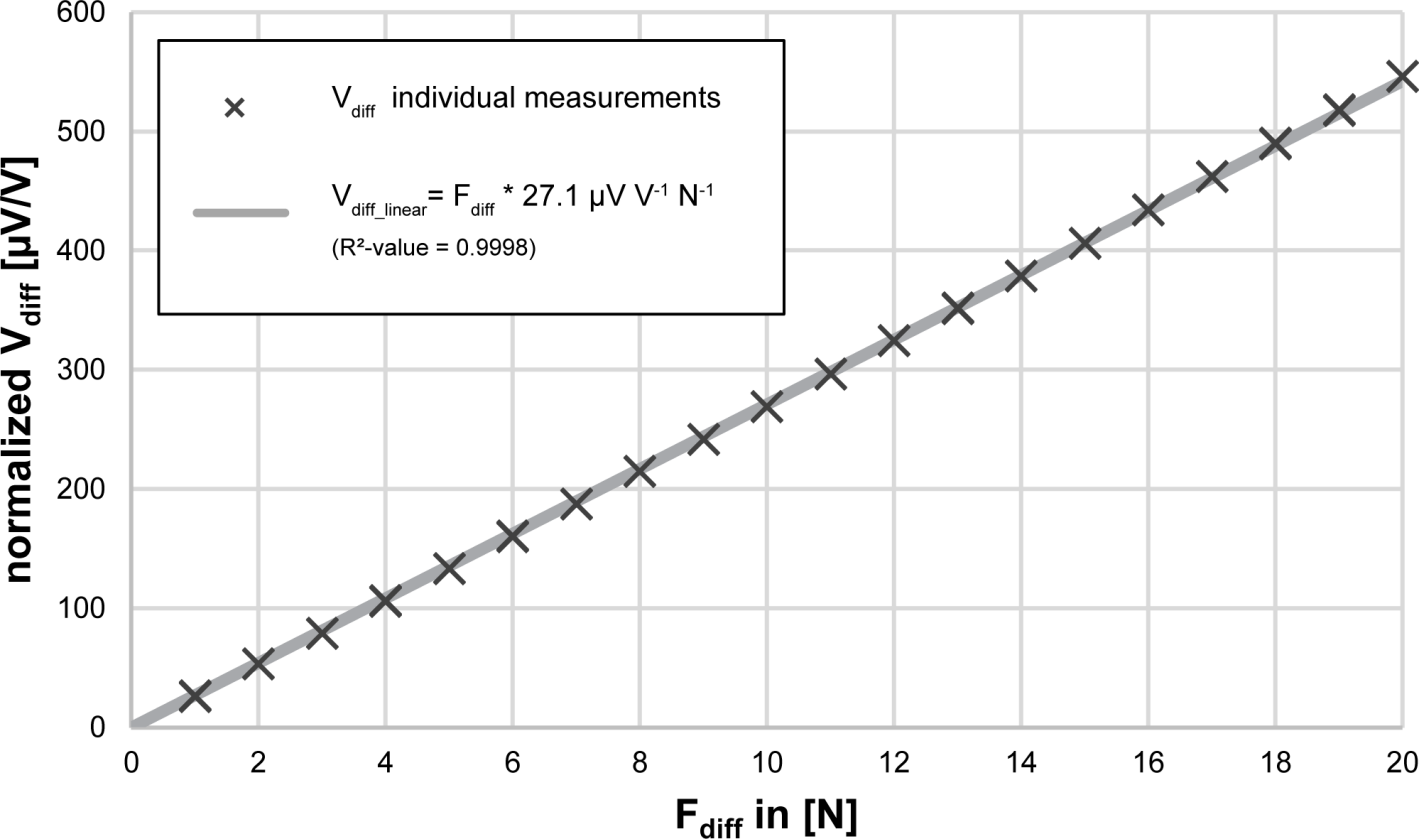
Calibration curve. Change in output-voltage (V_diff_) normalized to the bridge voltage (0.562 V) of the load-cell in relation to changes of force (F_diff_), measured with rectangular force pulses of different amplitudes. Crosses depict the results of the individual measurements for each force-level (amplitude). The individual measurements appear as single cross in the diagram due to the low variation. The solid grey line shows a linear regression performed on this data.

The calibration curve shown in Fig 5 depicts the individual measurements of V_diff_ obtained by the load-cell in correlation to the applied force-amplitude F_diff_. The load-cell shows a linear behavior that can be described as V_diff_linear_ = k * F_diff_ (k = 27.1 μV V^-1^ N^-1^) which fitted the measured data with a R^2^-value of 0.999 for the measured range of 0–20 N.

This linear regression was used as the calibration curve to calculate the corresponding force from the voltage signal measured with the load-cell.

Evaluating the difference between measured $\bar{V_{\mathrm{diff}}}$ and calculated V_diff_linear_ for every force level within the measured range, showed a maximal absolute linearity error of 3.9 μV at the highest applied force amplitude of F_diff_ = 20 N.

The highest standard deviation of the calculated means was found at F_diff_ = 18 N, amounting to 0.4 μV / V.

Relative errors were lower at higher force-levels as illustrated in Table 1. At full range (F_diff_ = 20 N) the maximal relative error was 0.07% of $\bar{V_{\mathrm{diff}}}$, while the standard deviation was ±0.04% of $\bar{V_{\mathrm{diff}}}$.

**Table 1:** Calibration curve measurements. Measurement results presented as mean voltage-difference ($\bar{V_{\mathrm{diff}}}$) and standard deviation (SD). The spreading of the data is reported as relative difference to $\bar{V_{\mathrm{diff}}}$ of the minimal and the maximal individual measurement of V_diff_ for each force-level. Further data of the overall linear regression (V_diff_linear_) and the corresponding absolute linearity error ($\bar{V_{\mathrm{diff}}}$– V_diff_linear_) are illustrating the load-cell´s linearity.

F_diff_	$\bar{V_{\mathrm{diff}}}$	SD	Maximal negative difference to $\bar{V_{\mathrm{diff}}}$	Maximal positive difference to $\bar{V_{\mathrm{diff}}}$	V_diff_linear_	Absolute linearity error
N	μV/V	μV/V	%	%	μV/V	μV/V
0	0	0.00	0.00	0.00	0.0	0.0
1	26.2	0.21	-1.59	0.94	27.1	-0.9
2	52.9	0.16	-0.48	0.56	54.2	-1.3
3	79.0	0.20	-0.61	0.23	81.3	-2.3
4	105.8	0.32	-0.32	0.67	108.4	-2.5
5	132.8	0.19	-0.21	0.18	135.5	-2.7
6	159.9	0.32	-0.33	0.32	162.6	-2.7
7	187.1	0.26	-0.19	0.26	189.7	-2.6
8	214.6	0.15	-0.11	0.12	216.8	-2.1
9	241.5	0.28	-0.16	0.21	243.9	-2.4
10	268.7	0.24	-0.11	0.20	271.0	-2.3
11	296.4	0.14	-0.08	0.02	298.1	-1.6
12	324.2	0.17	-0.10	0.08	325.2	-1.0
13	351.3	0.22	-0.09	0.12	352.3	-1.0
14	378.3	0.26	-0.10	0.15	379.4	-1.1
15	406.0	0.23	-0.08	0.07	406.5	-0.5
16	434.1	0.21	-0.06	0.07	433.6	0.6
17	462.0	0.25	-0.05	0.11	460.7	1.3
18	489.6	0.40	-0.12	0.16	487.8	1.8
19	517.5	0.24	-0.06	0.11	514.9	2.7
20	545.9	0.20	-0.05	0.07	542.0	3.9

### Step response

**Fig 6:**
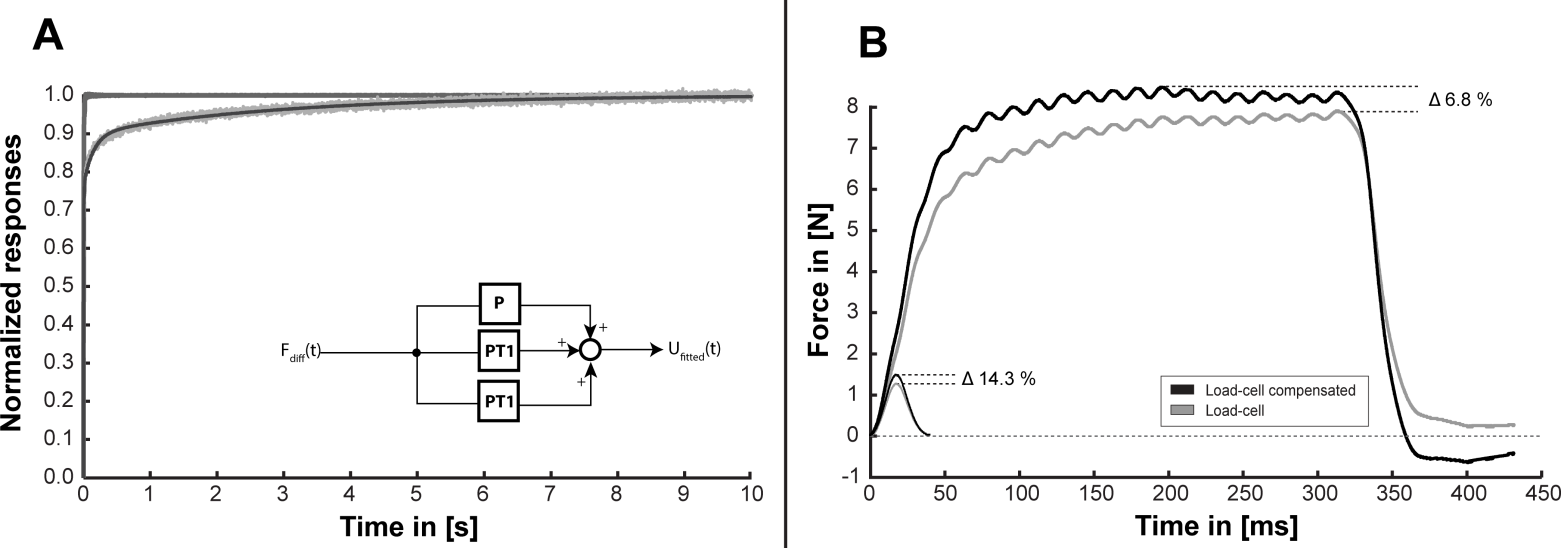
Step response. A) Illustration of the normalized signals measured with the reference sensor (dark grey trace) and the load-cell (light grey trace). The black trace represents a fitting (R^2^ = 0.977) to the normalized load-cell signal, considering the normalized reference-signal as input. The model of the load-cell is represented schematically as block-diagram of an additive combination of a P and two PT1 elements with the overall behavior of a PD2T2 element. B) The inverse transfer function of the PD2T2 model was used to correct the force response of a single twitch and a short burst (stimulation frequency 60 Hz). The exemplary data reveals an underestimation of the measured peak-forces by 14.3% for single twitches and 6.8% for short bursts.

The step response of the load-cell was measured with a rectangular 20 N force step. With respect to the maximal measured value, the response reached 25% after 2.9 ms, 50% after 5.9 ms and 75% after 20.6 ms. The step response was modeled using an additive combination of a P and two PT1 elements. The normalized signals of the load-cell and the reference-sensor are shown in Fig 6A. The proposed model fitted the measured data with an R^2^—value of 0.977. The corresponding parameters were k_0_ = 0.76 for the P-element; k_1_ = 0.13 and T1_1_ = 0.15 s for the first PT1-element and k_2_ = 0.10 and T1_2_ = 2.92 s for the second PT1-element. Calculating the parameters for an equivalent PD2T2 –element, characterizing the overall transfer-function of the load-cell revealed following values: k_P_ = 1, T_A_ = 2.62 s, T_B_ = 0.13 s, T_C_ = 0.15 s and T_D_ = 2.92 s. The inverse transfer function of the PD2T2 model could be used to compensate for the load-cell´s behavior, as demonstrated in Fig 6B by using Matlab´s function lsim.

### Hysteresis

**Fig 7:**
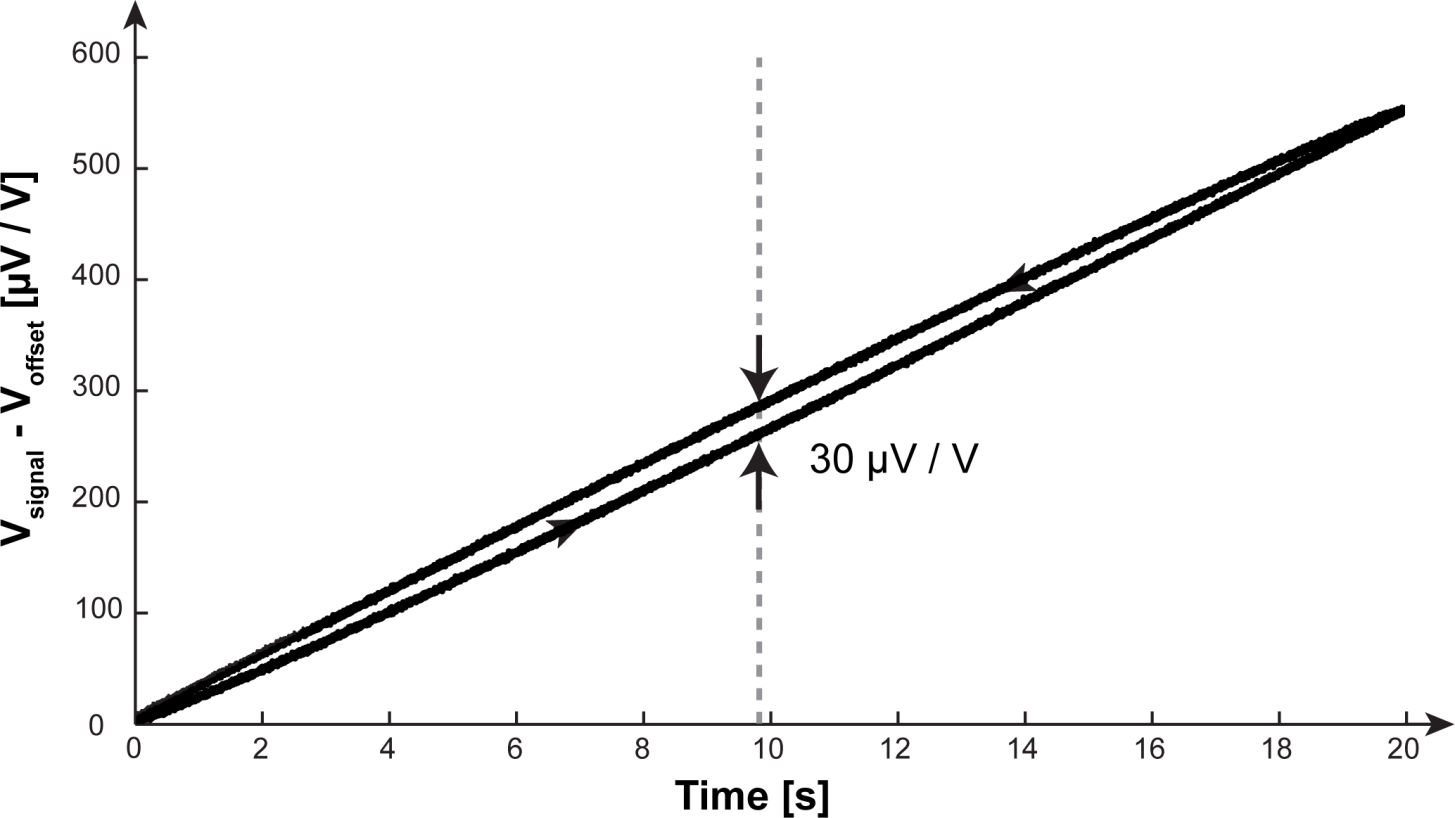
Hysteresis. Hysteresis was obtained by superimposing four consecutive responses, to a triangular force-pattern, measured with the load-cell. The hysteresis shows its highest width at 9.84 N which amounted to 30 μV / V.

The hysteretic behavior of the load-cell, for a linear rise of applied force from 1 to 21 N over a duration of 20 s and an equivalent linear decrease, is demonstrated in Fig 7. The maximal width of the hysteresis was located at 9.84 N and amounted to 30 μV / V.

### Frequency response

The mean peak-to-peak amplitude was evaluated separately for the signal of the load-cell and the reference sensor for frequencies of ranging from 0.01 to 200 Hz.

Within this range we did not observe any resonance effects, or signal overshooting. Frequencies higher than 140 Hz revealed a measured attenuation about -4 dB using the load-cell. The maximal phase delay of the load-cell, amounted to 8° at a frequency of 20 Hz. Our measured data (magnitude of amplitude damping, maximal phase delay) suggests that the resonance frequency of the sensor must be >200 Hz. The theoretical resonant frequency of a steel bar of these dimensions, given the velocity of sound in steel of (about 6000 ms^-1^) can be expected to be above 400 kHz (F_0_ = v / 2*L = 6000 ms^-1^ / 2*0.007 m = 428 kHz).

**Fig 8:**
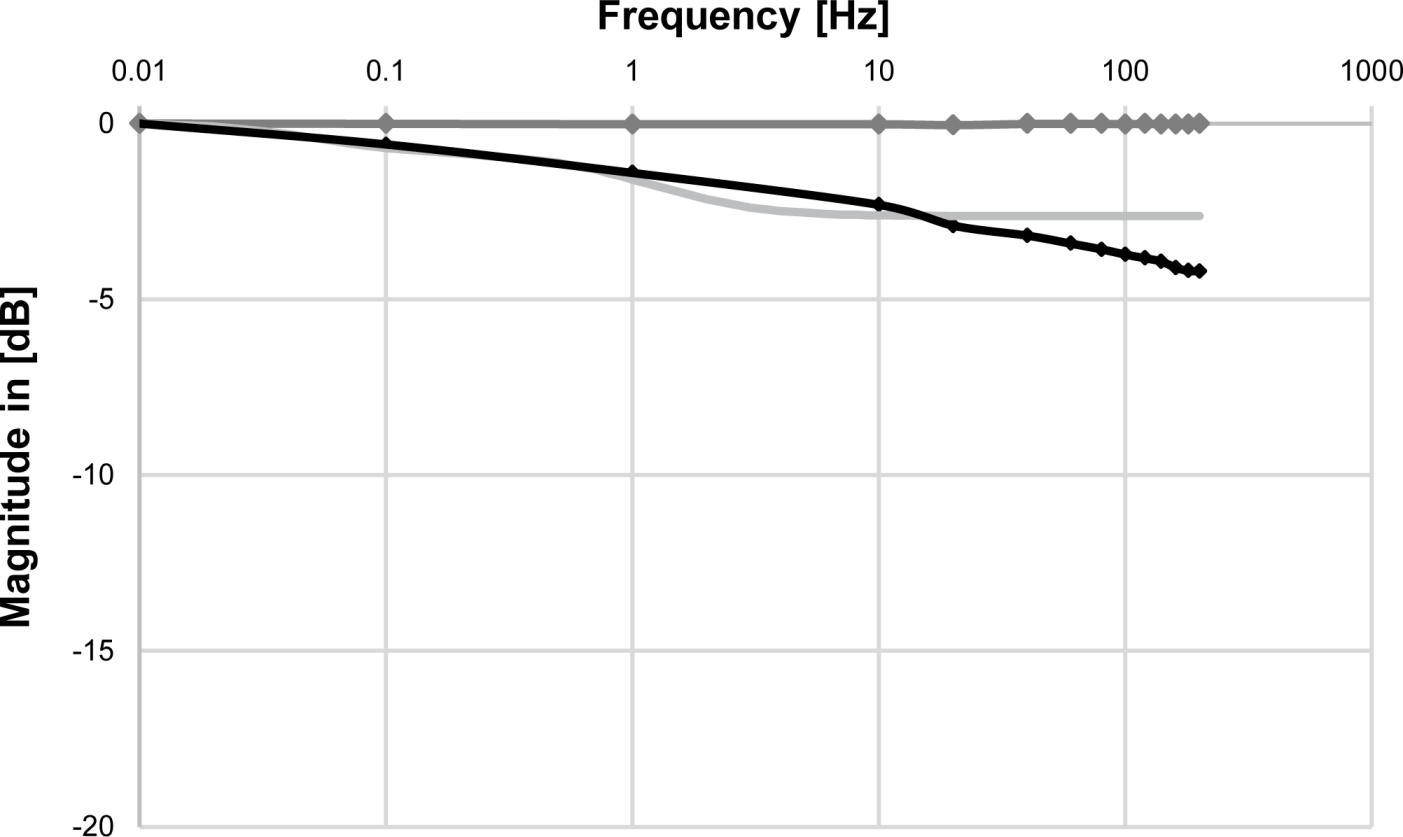
Frequency response. Frequency attenuation of the signals measured with the reference-sensor (dark grey trace) and the load-cell (black trace), in combination with the attenuation predicted by the calculated model of the load-cell (light grey trace). The model fits the measured load-cell data well at lower frequencies. For frequencies higher than 10 Hz a further decrease is observed in the measured data, revealing a maximal attenuation of -4.2 dB at a frequency of 200 Hz.

Data obtained with the reference-sensor did not show any attenuation (> -0.05 dB). Fig 8 shows a comparison between the frequency behavior predicted by the model (PD2T2-Element) and the actual measured values, along with the F_PP_ data evaluated for the reference sensor. The model correlates to the measured load-cell data up to a frequency of 10 Hz. The maximal difference between model and measured data amounted to 1.9 dB located at a frequency of 200 Hz.

### Reproducibility

To test the reproducibility of the load-cell´s output, a weight of 1 kg (9.81 N) was measured on 5 different days. The average voltage difference of a series of 5 consecutive loading cycles was used to estimate the accuracy of the load-cell. All measured values for V_diff_ were in the range of 254.8–282.1 μV / V. According to the linear calibration curve a V_diff_ of 265.8 μV / V could be expected when applying a weight of 9.81 N. The absolute deviation from this expected value ranged from -10.9 to 16.3 μV / V, which can be considered as a worst-case marker for the load-cell´s ability to predict an applied change in force. Using the calibration curve to calculate the corresponding force-level gives an estimated accuracy of 0.6 N (6.1%) for the middle of the measurement range.

The mean voltage difference and standard deviation can be found in Table 2 for each measurement day. The maximal observed standard deviation of 7.63 μV / V can be assumed as representative for the maximal variation of the sensor voltage for repeated measurements. By using the linear calibration curve, the load-cell´s precision can be estimated to 0.3 N (2.9%) for the middle of the measurement range. The corresponding 95% confidence interval of the slope, estimated based on the measurements with the highest variation, amounted to 2.5% of the regression slope of the calibration curve.

**Table 2:** Reproducibility. An 1 kg (9.81 N) weight was used on 5 different days to test the practical reproducibility of the load-cell’s. On each day the load-cell measured 5 consecutive loading/unloading cycles. The active force (V_diff_ = V_max_−V_offset_) was determined for each loading. Values expressed as voltage-difference ($\bar{V_{\mathrm{diff}}}$) and standard deviation (SD).

		Day 1	Day 2	Day 3	Day 4	Day 5
$\bar{V_{\mathrm{diff}}}$ (± SD)	μV / V	271.6 ± 5.51	263.1 ± 6.95	274.4 ± 7.63	255.5 ± 0.67	259.0 ± 1.34

## Discussion

The newly developed load-cell gives reproducible measurements of tensile forces of up to 20 N. This was verified by applying various patterns of forces followed by an analysis of the data recorded by the load-cell in comparison to a reference sensor. The measured shortening of the tendon, caused by the mandatory drying process of the small part of the tendon at the interface between tendon and load-cell, amounted to 3.6%, which can be considered to have a minor effect on the path of force transmission. Drying the tendon and attaching a solid force measurement device might alter the elastic properties of the biomechanical system. The load-cell was intended to be positioned close to the muscle, in a way that neither the load-cell nor the stiffer dried sections of the tendon will move across the ankle joint during movement. Therefore the natural movement of the foot will not be impeded, although the lower elasticity might cause a slightly different series elastic component of the muscle tendon complex. A slightly altered dynamics of force transmission which must be considered as a technical limitation of the device. The same limitation applies to the use of fixed force transducers where the tendon is clamped for attachment.

The load-cell itself showed a linear behavior between applied force and sensor voltage. The load-cell´s responses to rectangular force pulses, were repeatable and correlated very well (R^2^ = 0.999) with the amplitude of the applied force-pulses. Comparable force-transducers [15,18,23] report their correlation-coefficients to be greater than 0.95–0.99. The stated values for accuracy (±6.1%) and precision (±2.9%) for the middle of the measurement range, should be interpreted as practical estimates according to the data measured with the load-cell. These values match our initial expectation which we consider adequate to estimate the type and amount of loading experienced by the TA-muscle in the intact rat. As expected for this type of sensor the highest relative errors were seen at the lower end of the measurement-range. Additionally, at low force levels accuracy and precision are influenced by the resolution of the measurement system, which according to the manufacturer’s specification is 625 nV (equivalent to 0.06 N) for the selected input voltage range of 20 mV. Buckle force transducers [15,23,24] have been reported with 95% confidence intervals of the slope below 2 to 4% of the regression slope. Based on our reproducibility measurements with the load-cell, the 95% confidence interval of the slope amounted to 2.5% which is comparable to other systems.

Analysis of the response of the load-cell to a 20 N step increase in force showed a steep proportional rise in the beginning, followed by a much slower creep-like rise—revealing the behavior of a PD2T2-element. The proportional part is explained by the expected deformation of the stainless steel core, while the creep-like part may be caused by the instant-adhesive used to encapsulate the strain-gauges. Creeping-effects are unfavorable effects frequently seen in different materials during mechanical loading. In metals these effects are usually rather slow, occurring at high temperatures within a timescale of hours or days [25,26]. Polymers on the other hand reveal a faster time-constant in the range of several seconds to minutes [27], which could explain the observed behavior of the load-cell considering the instant-adhesive as an additional load-bearing element. With respect to the measured value of the step-response after 2 s, which was used to create the calibration curve, it could be expected that the load-cell underestimates forces produced during short bursts (300 ms) by 8% or twitches (rise time ~20 ms) by 19%. As long as the load-cell operates within its linear region (F_diff_ < 20 N), the inverse transfer function of the PD2T2 model can be used to compensate for the sensor’s behavior, as demonstrated in Fig 6B. This compensation does not affect our calculations of accuracy or precision. We defined accuracy as maximal deviation from the value expected from our calibration curve using static loads. We further defined precision to be the maximal standard deviation experienced within one measurement set (measurement day). As the inverse transfer function only corrects for the shape of the step-response, accuracy and precision will stay the same. For routine use, for example comparing relative peak forces during fused contractions, the correction based on the inverse dynamics model would not be necessary. If a study focused on dynamic responses, then transformation of the data according to the model of transducer behavior in step responses could be considered.

The model fits very well in the range below 10 Hz. Fig 8 shows that an additional low pass cut off frequency between 10 and 100 Hz could be added to upgrade the model to PD2T3. This time constant is hard to extract from the step response in the time domain since the PD2T2 model already fitted with R^2^ = 0.977. If a model of higher order is required the additional cut off frequency must be estimated in the frequency domain.

The hysteresis can be interpreted as a direct consequence of the behavior observed for the load-cell´s step response. The hysteretic width describes the signal difference, when approaching a particular force-level either from the upper or lower end of the measurement range. Thus, the maximal hysteretic width indicates the maximal hysteresis error (5.4% of the full-range signal) that can be expected for the load-cell.

The difference in the attenuation of force predicted by the model and measured data from the load-cell, fits very well for lower frequencies while deviation is observable at higher frequencies. The maximal attenuation seen at 200 Hz, led to a ~4 dB lower voltage than at 0.01 Hz. In the context of muscular forces this attenuation is of minor concern as the fusion-frequency of muscles is generally much lower, meaning that higher frequency components only having a minor contribution to the overall measured force signal. In the case of single twitches one might anticipate problems due to steep rising times. However, the isometric contraction-time for single twitches of the tibialis anterior muscle in healthy rats is reported between 20.3 ms [9] and 32 ms [6] with maximal twitch peak-forces of 2.31 N and 1.41 N, respectively. In linear approximation this would correspond to a steepness ranging from 44 to 114 N s^-1^. The based on the rising time of the initial 25% (equivalent to 5 N) rise of the load-cell’s step-response is at 1698 N s^-1^. The response of the load-cell is therefore fast enough to measure single twitches. Attenuation is constant regardless of the measured twitch-amplitude and will not influence the outcome of comparative measurements like recruitment-curves or measurements to test different regimes of muscle loading.

The load-cell has proven to be a useful force measurement device which has already been used in a series of 11 individual experiments performed on male adult Wistar rats, measuring tensile forces up to 16.5 N [28]. The ability to measure the force of the muscle while still acting in its natural biomechanical environment provides valuable additional information compared to measurements in which the muscle is isolated from its original attachments. The outcome of such measurements performed during acute experiments is of particular interest in the planning stage when designing experimental protocols to investigate the adaptive response of muscle to changes in loading. For example, studies with programmed muscle activation via implanted nerve stimulators over a period of a few weeks [1,2,12] or studies examining the response of bone to the stresses imposed by programmed muscular contractions [8]. Force that is transmitted through the tendon via the ankle joint could be used to train the tibialis anterior in various different ways, for example by imposing extra resistance with co-contraction of antagonistic muscles. To design a rational training regime it is important to estimate the force profile experienced by the muscle of interest—in order to select a training stimulus that is challenging enough to trigger muscular adaptations such as muscle hypertrophy without causing irreversible damage to the muscle. Acute experiments under anesthesia using the described in-line load-cell can inform the design of long-term experiments in which muscles are activated and loaded over periods of days or weeks by means of implanted stimulators in freely-moving animals.

## Conclusions

We have shown that the newly developed load-cell is able to measure tensile forces of up to 20 N, without inelastic deformation of the sensor. Due to its small dimensions, the load-cell qualifies for various applications in which it is of interest to directly measure the tensile forces within a particular tendon without major rearrangement of the biomechanical system. The load-cell has already been used to perform functional measurements in rats to assess the forces generated by the TA-muscle as well as forces imposed on the TA-muscle by contraction of the plantar flexor muscles. Such forces are transmitted across the ankle joint and therefore can only be measured if the muscles continue to act on their original points of insertion. These measurements provide valuable guidance in the design of training protocols for the TA-muscle.
